# Effect of Post-Thermal Annealing on the Performance and Charge Photogeneration Dynamics of PffBT4T-2OD/PC_71_BM Solar Cells

**DOI:** 10.3390/polym11030408

**Published:** 2019-03-02

**Authors:** Wei Zhang, Rong Hu, Xiaokang Zeng, Xiaojun Su, Zhifeng Chen, Xianshao Zou, Jun Peng, Chengyun Zhang, Arkady Yartsev

**Affiliations:** 1 School of Physics and Electronic Engineering, Guangzhou University, Guangzhou 510006, China; chenzf@gzhu.edu.cn (Z.C.); speepengjun@gzhu.edu.cn (J.P.); chyzhang@gzhu.edu.cn (C.Z.); 2 Research Institute for New Materials Technology, Chongqing University of Arts and Science, Chongqing 402160, China; xiaokang_zeng@126.com (X.Z.); 3 NanoLund and Division of Chemical Physics, Lund University, Lund 22100, Sweden; xiaojun.su.6673@chemphys.lu.se (X.S.); xianshao.zou@chemphys.lu.se (X.Z.); Arkady.Yartsev@chemphys.lu.se (A.Y.)

**Keywords:** polymer solar cell, power conversion efficiency, charge generation, thermal annealing

## Abstract

In this work, we studied influence of post thermal annealing on the performance and charge photogeneration processes of PffBT4T-2OD/PC_71_BM solar cells. As-prepared device exhibits a high-power conversion efficiency of 9.5%, much higher than that after thermal annealing. To understand this phenomenon, we studied charge photogeneration processes in these solar cells by means of time resolved spectroscopy. We associate the degradation of solar cell performance with the reduction of exciton dissociation efficiency and with increased bimolecular recombination of photogenerated charges as a result of annealing. We correlate the generation of localized PffBT4T-2OD polarons observed via spectro-electrochemical measurements with enhancement of the bimolecular charge recombination of annealed solar cells.

## 1. Introduction

**Figure 1:**
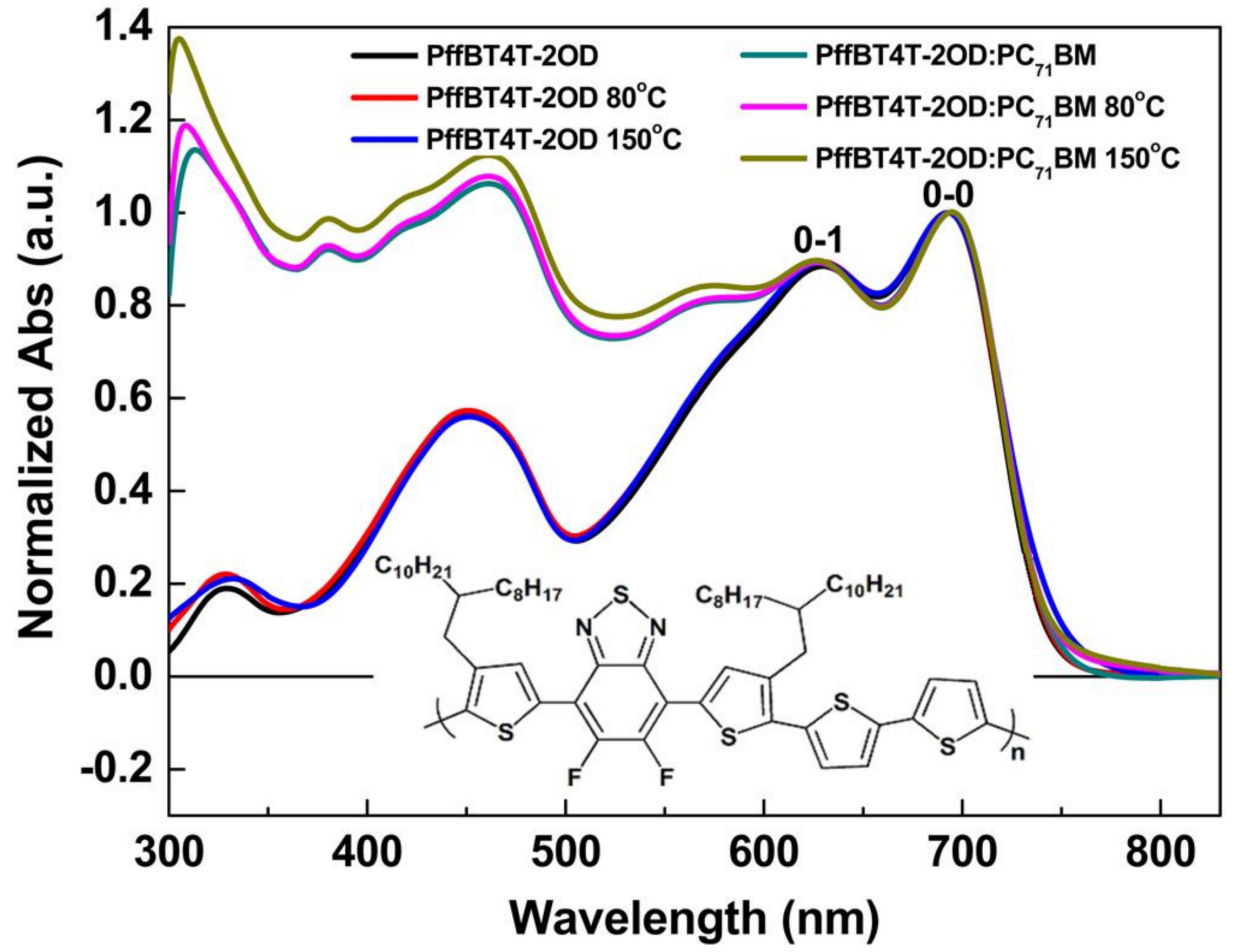
Normalized absorption spectrum of neat PffBT4T-2OD and PffBT4T-2OD:PC_71_BM blend films at the indicated processing conditions. Inset is the chemical structure of PffBT4T-2OD.

Polymer solar cells belong to the third-generation solar cell with a number of advantages such as light weight and flexibility, low cost, easy processing for mass production and so on [1,2,3,4]. Over the past decade, polymer solar cell developed very fast and the power conversion efficiencies (PCE) of single junction and tandem solar cells has exceeded 14% [5,6,7] and 17% [8], respectively, owing to the development of active layer materials, design of electrode, etc. Poly[(5,6-difluoro-2,1,3-benzo-thiadiazol-4,7-diyl)-alt-(3,3′′′-di(2-octyldodecyl)-2,2′;5′,2′′;5′′,2′′′-quaterthiophen-5,5′′′-diyl)] (PffBT4T-2OD) (Figure 1) is a crystallizable polymer with a high hole mobility, which can be manipulated to form an optimal structure for charge photogeneration in bulk heterojunction solar cells [9,10,11,12,13,14,15].

Currently, PCE of PffBT4T-2OD based binary and ternary solar cells has exceeded 10% [9,10,13,15]. In these studies, it has been claimed that the interface engineering is required to achieve high PCE to control the morphology of active layer. Thermal annealing is an important method for regulating the morphology of bulk heterojunction polymer solar cells, and has become an essential option for optimizing the device efficiency and performance. Thermal annealing was also applied for fabricating PffBT4T-2OD/PC_71_BM solar cells [9,10,11,12,13,14,15], providing high efficiency solar cells [9,10,13,15]. However, the degradation of PffBT4T-2OD/PC_71_BM solar cell performance with post thermal annealing was also reported [16]. Despite thorough studies of the influence of post annealing on the performance of PffBT4T-2OD/PC_71_BM solar cells, the correlation between the device performance and the dynamics of photogenerated charges is not settled yet. Understanding these correlations is essential for optimizations of the performance of PffBT4T-2OD-based solar cells.

In this work, we have studied an effect of thermal annealing on the performance and charge dynamics in PffBT4T-2OD/PC_71_BM solar cells. We combined time resolved photoluminescence and transient absorption to examine the dynamics of PffBT4T-2OD exciton dissociation and carrier recombination processes in PffBT4T-2OD/PC_71_BM blend films. Further, we discuss the correlation between the device performance and photogenerated charge dynamics. With the help of the time resolved photoluminescence measurements, we find that thermal annealing reduces exciton dissociation rate and thus exciton dissociation efficiency. In transient absorption study, we conclude that thermal annealing facilitates bimolecular recombination of the photogenerated charges. To explore the details of the carrier recombination process and to analyze the composition of charges in PffBT4T-2OD/PC_71_BM films with and without thermal annealing, spectro-electrochemical spectra measurements were conducted. We find that thermal annealing facilities generation of the localized PffBT4T-2OD polarons, which may lead to enhancement of bimolecular charge recombination. Higher exciton dissociation rate and lower bimolecular recombination result in a higher power conversion efficiency of unannealed device comparing to the thermal annealed ones.

## 2. Experimental Section

### 2.1. Sample Preparation

Poly[(5,6-difluoro-2,1,3-benzothiadiazol-4,7-diyl)-alt-(3,3′′′-di(2-octyldodecyl)-2,2′;5′,2′′;5′′,2′′′-quaterthiophen-5,5′′′-diyl)] (PffBT4T-2OD) and [6,6]-phenyl-C_71_-butyric acid methyl ester (PC_71_BM) were purchased from Solarmer Energy Inc. Solar cells were fabricated using an inverted device configuration of indium tin oxide (ITO) electrode/zinc oxide (ZnO) layer/active layer/molybdenum oxide (MoO_3_) layer/Ag electrode. The ITO substrate was pre-cleaned with detergent, deionized water, acetone, chloroform and isopropyl alcohol under the ultrasonic condition, and was dried in a heat oven at 80 °C. The ZnO layer was prepared from a precursor solution, which contained zinc acetate (1 g), 2-methoxyethanol (1 mL) and ethanolamine (0.28 mL). The precursor solution was spin-coated onto ITO substrate and then annealed at 200 °C for 60 min in air. PffBT4T-2OD and PC_71_BM were dissolved in a mixed solvent (CB:DCB = 1:1 with 3% of 1,8-diiodooctane). The concentrations of PffBT4T-2OD and PC_71_BM in the mixed solutions were 10 and 12 mg/mL, respectively. The mixed solutions were heated at 100 °C on a hot plate for 6 h. After that, the mixed solutions (~100 °C) were quickly transferred onto the substrates with hot ZnO layer which was pre-heated at ~100 °C (i.e., the ZnO/ITO substrate was heated by a hot plate at 100 °C first, and then transferred onto the spin-coater quickly) and spin-coated at a speed of 800 rpm for 10 s. After that, the active layers were annealed at varied temperatures (0 °C, 80 °C and 150 °C) for 5 min. Preparation of the polymer solutions, spin-coating and thermal annealing of the active layer were all conducted in a glovebox filled with N_2_. Then, 8 nm MoO_3_ and 80 nm Ag electrode were sequentially deposited on the surface of active layer using a shadow mask to obtain an effective area of the cell (0.03 cm^2^) and form a top anode in a vacuum chamber (below 9 × 10^−5^ Pa) integrated with a glovebox.

### 2.2. J-V Measurement and Structure Characterization

Current density-voltage (*J-V*) characteristics of the devices was recorded by a computer controlled Keithley 2400 source meter under the illumination of an AM 1.5G solar simulator (Model 7IS0503A, Sofn Instruments Co. Ltd., Beijing, China) with an intensity of 100 mW/cm^2^. The intensity of the light was determined using a standard silicon solar cell (Model 7-SSC10, Sofn Instruments Co. Ltd., Beijing, China). *J-V* characteristics were acquired for different solar cells, each at a separate substrate. Morphology of the active layer of each solar cell was characterized by an atomic force microscopy (AFM, AFM-5500, Agilent Technologies Inc., Palo Alto, CA, USA) working in the tapping mode. *J-V* and AFM measurements were done at room temperature in air.

### 2.3. Spectroelectrochemical (SEC) Measurement

SEC measurements were conducted by means of a three-electrode system with a working electrode (ITO with active layer), a counter electrode (platinum wire) and a reference electrode (Ag+/Ag, 0.01 M Ag+ in acetonitrile) [17]. The electrolyte solution for these measurements was tetra-*n*-butylammonium hexafluorophosphate (0.1 M) in acetonitrile. All the electrodes and electrolyte solutions were assembled in a quartz cell, which was placed in a UV-Vis-NIR spectrophotometer (Cary5000, Agilent Technologies Inc., Palo Alto, CA, USA) for measurements. The applied electrical potential across the reference and the working electrodes was 1.2 V, which is higher than the onset of the oxidation potential of PffBT4T-2OD [10] and can promote generation of PffBT4T-2OD^+^. The SEC spectra were taken as the difference between the absorption spectra with and without the applied potential. All measurements were conducted in air at room temperature.

### 2.4. Time-Resolved Photoluminescence Measurements (TRPL)

TRPL was measured in a setup described in Reference [18]. The frequency-doubled (400 nm) *s*-polarized light from Ti:Sapphire fs laser (Spectra-Physics Tsunami, MKS instruments Inc., Andover, MA, USA) with a pulse duration of ~100 fs was used for excitation. PL was collected and focused on the input slit of a spectrograph (Model 250is, Chromex Inc., Albuquerque, NM, USA) by two quartz plano-convex lenses. The output of the spectrograph was projected onto the input slit of the streak camera (Hamamatsu C6860, Hamamatsu Photonics, Hamamatsu, Japan). All samples were kept in N_2_ and were measured at room temperature. Efforts were taken to keep the alignment of the experimental set-up the same for all the samples studied.

### 2.5. Time Resolved Transient Absorption (TA)

TA signal was measured in a setup described in Reference [19]. The output of a regenerative amplified femtosecond laser system (Pharos, Light Conversion, Vilnius, Lithuania) operating at 1030 nm and delivering pulses of 200 fs at a 2 kHz repetition rate was used to pump two non-collinear optical parametric amplifiers (Orpheus-N, Light Conversion, Vilnius, Lithuania). One of them was used to generate pump pulses centered at 650 nm. The second NOPA was used for probe pulses centered at 700 nm. The probe was delayed with respect to the pump by a mechanical delay stage. All samples were kept in N_2_ and measured at room temperature.

## 3. Results and Discussion

### 3.1. Steady State Absorption Properties

Figure 1 shows normalized optical absorption spectra of PffBT4T-2OD film and PffBT4T-2OD:PC_71_BM blend films at varied processing conditions. For the net PffBT4T-2OD film, thermal annealing does not lead to visible difference in the vibronic 0–0 band and 0–1 sub-bands within the manifold of the π–π* electronic transitions. For PffBT4T-2OD:PC_71_BM blend films, we find that absorption in the range of 300 nm–550 nm is significantly higher than that of the neat polymer film due to absorption of the PC_71_BM. It is known that for PffBT4T-2OD:PC_71_BM films, the relative amplitudes of 0–0 band and 0–1 band are sensitive to the spinning rates and the substrate temperature in the spin coating process [10], similar to what was also observed in classic P3HT:PC_61_BM films [20,21,22,23]. Herein, we note that the absorption spectrum of the films with and without annealing are similar, implying that thermal annealing has a weak influence on the absorption of the PffBT4T-2OD:PC_71_BM blended film.

### 3.2. Morphology Characterization

**Figure 2:**
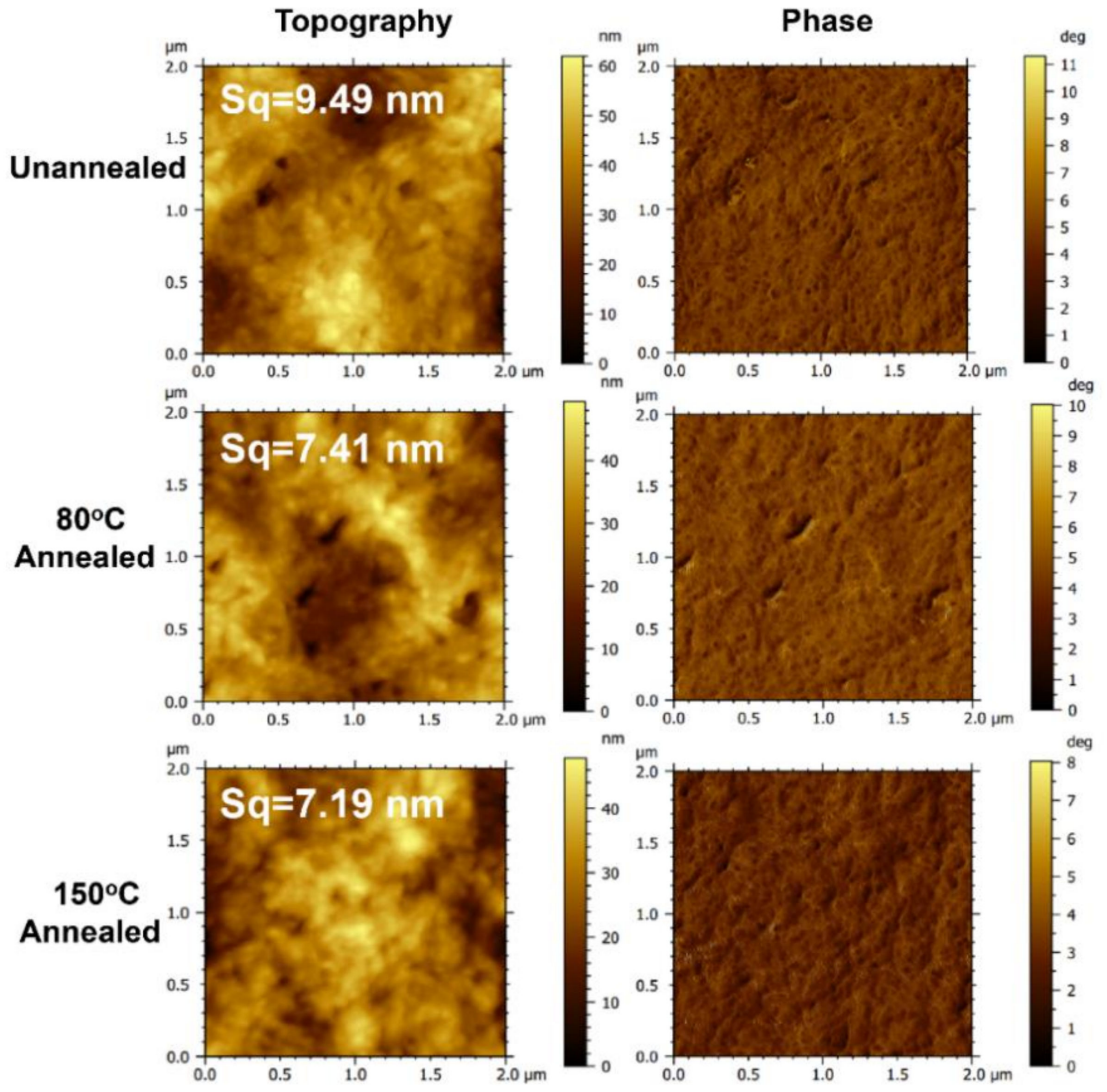
Representative AFM topography (2 × 2 µm^2^) and phase (2 × 2 µm^2^) of unannealed and annealed PffBT4T-2OD:PC_71_BM films.

The morphologies of PffBT4T-2OD:PC_71_BM blend films were examined by atom force microscopy (AFM) measurements, as shown in Figure 2. The surface roughness is usually expected to reflect the self-organization of polymers in the blend films, which can be promoted by annealing processes, such as solvent vapor annealing, thermal annealing, etc. [24,25,26,27]. Herein, we find that the root-mean-square of the surface roughness (Sq), which is equivalent to the standard deviation of the polymer surface from the substrate plane, decreases under annealing: unannealed (9.49 nm) > annealed (7.41 nm for 80 °C and 7.19 nm for 150 °C), implying that thermal annealing can smoothen the surface of PffBT4T-2OD:PC_71_BM blend films.

### 3.3. Photovoltaic Performance

**Figure 3:**
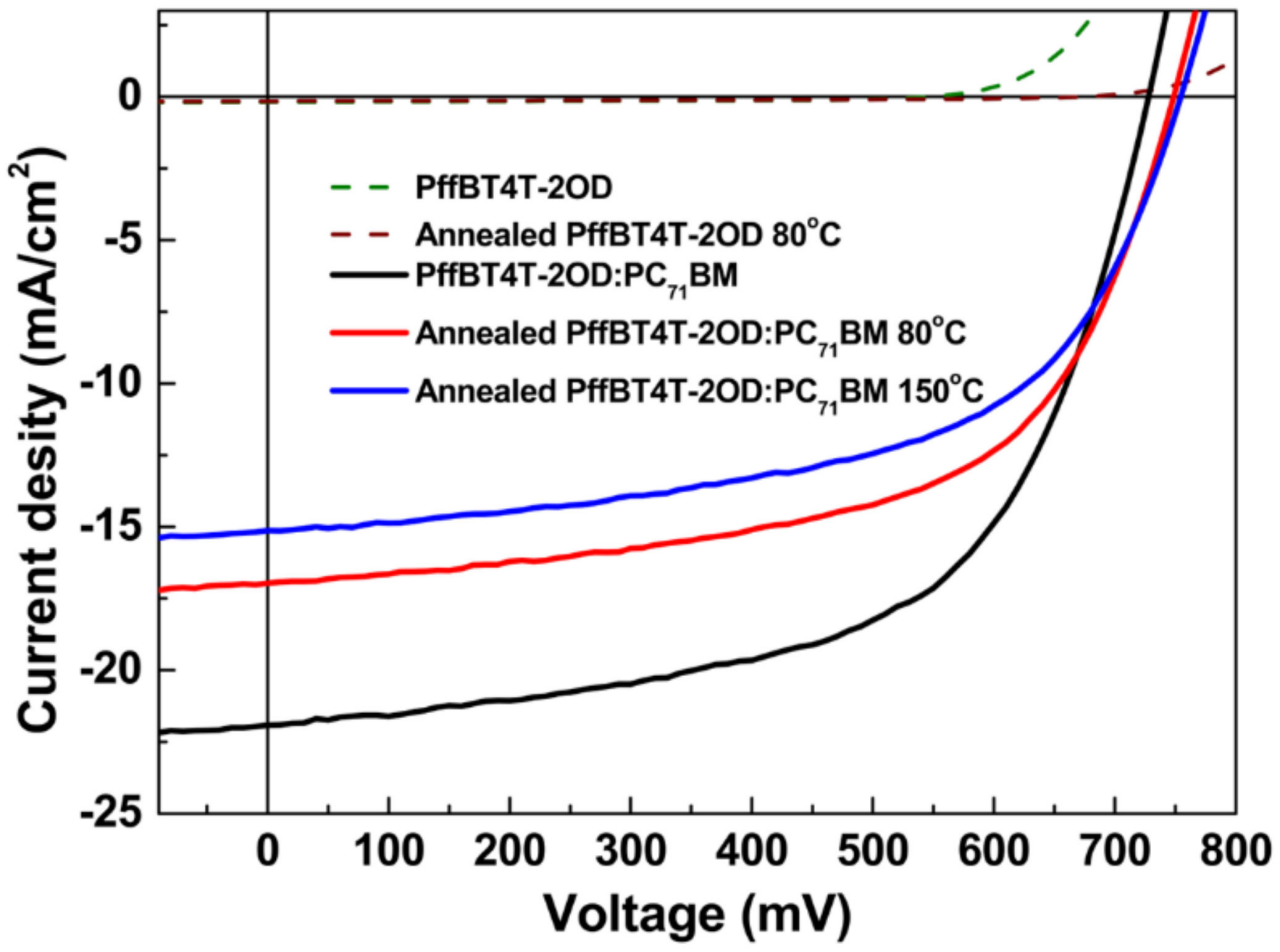
Averaged *J*-*V* characteristics of neat PffBT4T-2OD and PffBT4T-2OD:PC_71_BM solar cells. The *J*-*V* characteristics were averaged by 5 devices.

To study the effect of post annealing on the performance of PffBT4T-2OD:PC_71_BM solar cells, we compare averaged *J*-*V* properties of unannealed and annealed at 80 °C and 150 °C devices (Figure 3). The detailed *J-V* properties of each device can be found in Supplementary Materials S1. The corresponding device parameters are summarized in Table 1. The PffBT4T-2OD:PC_71_BM solar cells without annealing shows a PCE of 9.50% with a *V*_oc_ of 0.73 V, *J*_sc_ of 21.92 mA/cm^2^ and FF of 0.60. After thermal annealing, the devices (80 °C and 150 °C annealed) exhibit *V*_oc_ of ~0.75 V and 0.76 V, which are slightly higher than that for the unannealed device (0.73 V). This trend is in agreement with the previous reports of the thermal annealing effect on the PffBT4T-2OD:PC_71_BM solar cells [16]. However, compared to unannealed device, *J*_sc_ decreases significantly to 16.97 and 15.32 mA/cm^2^ for the devices annealed at 80 °C and 150 °C, respectively, and their FF have a slight reduction for all annealed devices. As a result, PCE decreases with annealing temperature. Thus, *J*_sc_ is the critical parameter that varies in the PffBT4T-2OD:PC_71_BM solar cells under annealing. Usually, *J*_sc_ is related to conversion efficiency of absorbed light photons into extracted charges. Thus, we employ time-resolve photoluminescence (TRPL) and transient absorption (TA) techniques to study exciton dissociation and carrier recombination processes as the key processes related to *J*_sc_.

### 3.4. Exciton Dissociation

**Figure 4:**
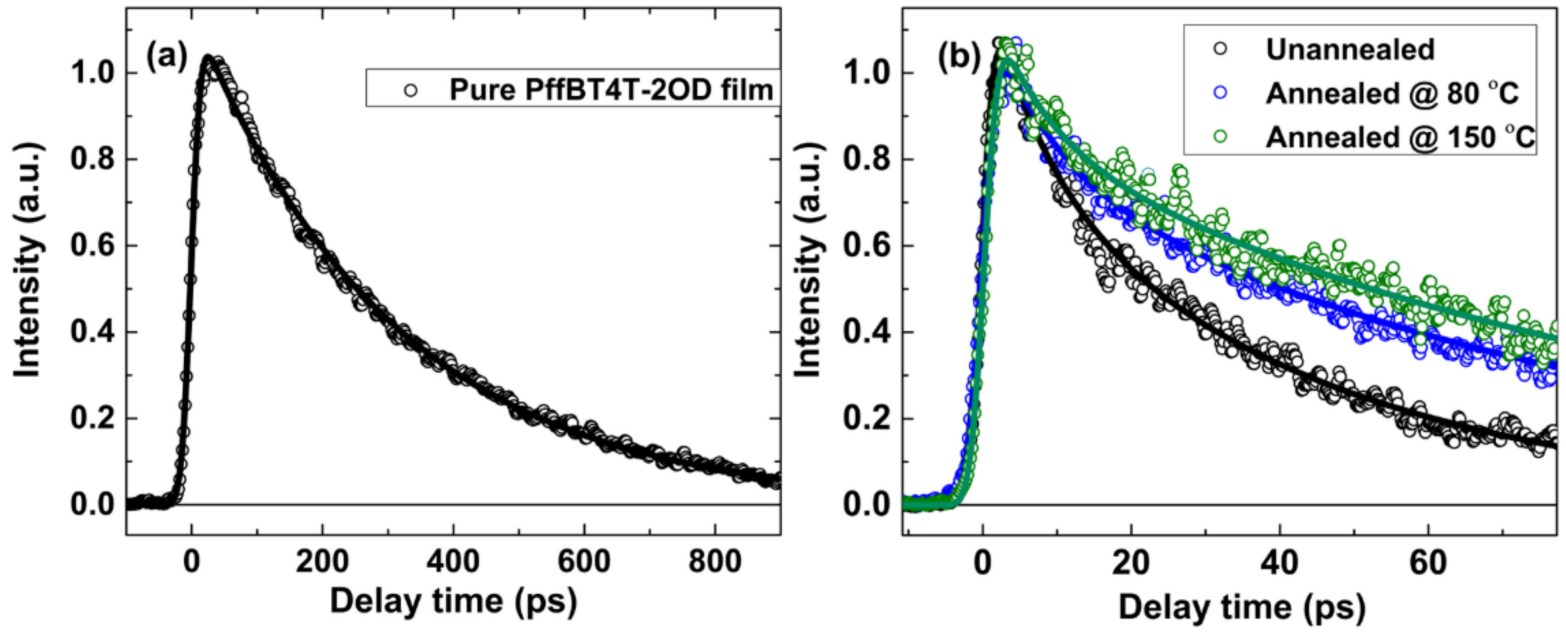
TRPL kinetics of (**a**) neat PffBT4T-2OD and (**b**) PffBT4T-2OD:PC_71_BM films after photoexcitation at 400 nm. The kinetics are fitted by exponential decay functions, and the fitting parameters are summarized in Supplementary Materials Table S1.

To gain insight into exciton diffusion and dissociation processes in blend films, we measured TRPL kinetics of neat PffBT4T-2OD and PffBT4T-2OD:PC_71_BM blend films, as shown in Figure 4. Samples were excited at 400 nm and emission at 810 nm was analyzed to study the dynamics of the PffBT4T-2OD excitons. For the neat PffBT4T-2OD film, kinetics exhibits a mono-exponential decay with a lifetime of ~305 ps. For the PffBT4T-2OD:PC_71_BM blend films, the kinetics decay much faster than in neat PffBT4T-2OD film, in agreement with the expectation that the PffBT4T-2OD excitons dissociate due to efficient charge generation after blending with PC_71_BM. We note that TRPL kinetics of the blend films are non-exponential. This non-exponential PL decay can be attributed to inhomogeneous size distribution of the donor phase, which has been observed in plenty of high-efficient solar cells. To quantify PL decay time of the samples, we define the time for the emission to decay to 1/e of its initial value as a PL lifetime. We find that PL lifetime of unannealed PffBT4T-2OD:PC_71_BM film (~35 ps) is shorter than that of the film annealed at 80 °C (~65 ps) and 150 °C (~77 ps). Assuming that the diffusion coefficient of the PffBT4T-2OD excitons is the same for all the blended films and assuming that the exciton dissociation probability at the PffBT4T:2OD:PC_71_BM interface does not vary after annealing, such longer PL decay lifetime indicates longer exciton diffusion length and thus correspondingly larger size of the PffBT4T-2OD domains. Accordingly, we conclude that thermal annealing increases the average size of the donor domains in the blend.

From the PL lifetimes of the neat PffBT4T-2OD and PffBT4T-2OD:PC_71_BM blended films, we estimate the PffBT4T-2OD exciton quenching efficiency in the blends using the exciton lifetime (τ) measured by TRPL via $\eta_{\mathrm{donor}}=1-\frac{\tau_{\mathrm{blend}}}{\tau_{\mathrm{pure}}}$ [28]. The estimated PffBT4T-2OD exciton dissociation efficiencies are ~89%, ~79% and ~75% for the unannealed, 80 °C and 150 °C annealed blends, respectively. Thus, we infer that thermal annealing reduces exciton dissociation efficiency of the donor in the blends due to the increase of the donor domain size. In principle, a suitable phase separation is needed for promoting charge transport and inhibiting the carrier recombination loss of polymer solar cells [29,30,31,32].

The decrease of exciton dissociation efficiency by ~10% and 14% as a result of annealing comprises a large part of the *J*_sc_ decrease after 80 °C and 150 °C thermal annealing of the PffBT4T-2OD:PC_71_BM blended films, respectively. Yet, another process has to be responsible for the rest of the *J*_sc_ decrease. In the following, we will analyze the dynamics of photogenerated charges to identify the other mechanisms responsible for the *J*_sc_ decrease.

### 3.5. Spectral Characterization of the PffBT4T-2OD Radical Cations in Neat and Blended Films 

**Figure 5:**
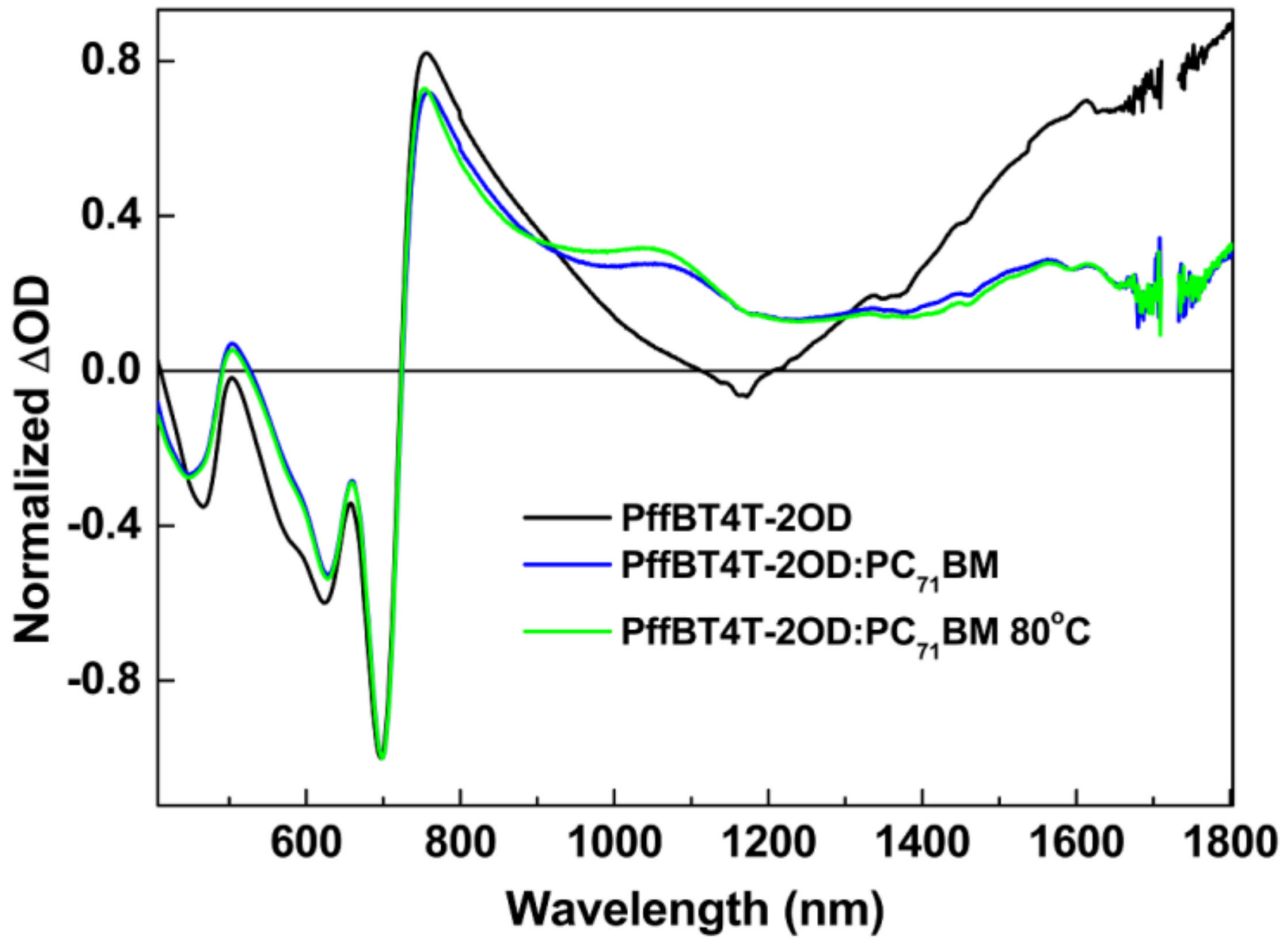
Normalized spectro-electrochemical spectra of the neat PffBT4T-2OD and PffBT4T-2OD:PC_71_BM blended films. The SEC spectra were taken as the difference between the absorption spectra with and without the applied potential (1.2 V).

To identify charge species in the blended films, spectro-electrochemical (SEC) measurements were performed on the neat and blended films, as shown in Figure 5. The SEC spectra of both neat and blended films exhibit the electrooxidation induced bleaching of the ground state absorption with the 450, 630, 700 nm negative bands. Additionally, to the red side of the ground state bleaching, we record the positive band with peaks at ~750 nm for both neat and blended films and ~1060 nm band visible only in the blended films. In analogy with the assignment of absorption spectra of charges in P3HT film [17,20,33,34], we attribute the 750 nm positive band of the SEC spectra to the delocalized polaron (DP) inhibiting at PffBT4T-2OD crystalline polymers whereas the 1060 nm band is associated to the localized polaron (LP) polarons residing on the disordered polymer molecules. We also observe a broad positive SEC band at the wavelength longer than 1200 nm, which can be attributed to the HOMO-to-SOMO electronic transition of the polarons [17,33,34]. Here, we can exclude that the influences of the recorded spectral signal originates from oxidation of the PC_71_BM as we do not observe any spectral characteristics of the ground state bleaching corresponding to PC_71_BM in the range of 330 nm ~ 550 nm [35].

**Scheme 1:**
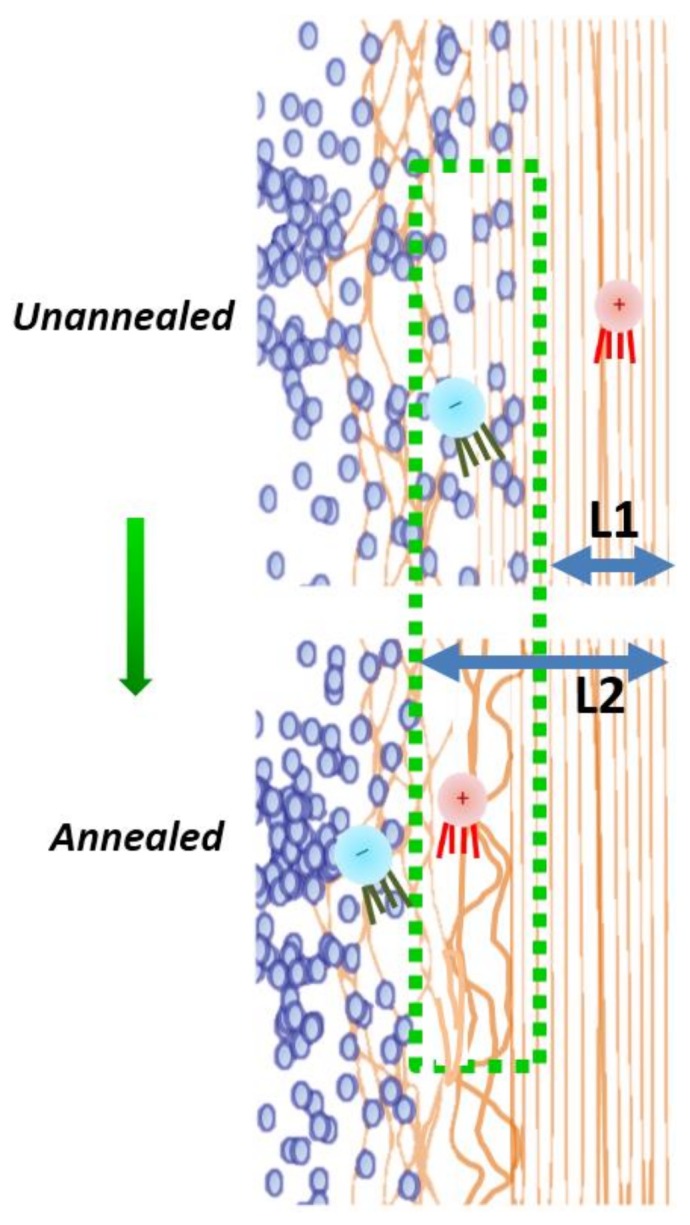
Schematic illustration of the morphology evolution of the PffBT4T-2OD:PC_71_BM blends after post thermal annealing. After thermal annealing, the originally ordered PffBT4T-2OD around the interface (dotted line region of unannealed sample) was disarranged at the interface (dotted line region of annealed sample), due to the aggregation of the PC_71_BM. Meanwhile, the size of the pure PffBT4T-2OD phase increases from L1 to L2, due to the aggregation of the donor during thermal annealing process.

We note that population of LPs (~1060 nm band) in the neat film is negligible comparing to that of DPs (~750 nm band), suggesting that DP is preferentially generated in the neat PffBT4T-2OD film. Furthermore, the population of LPs (~1060 nm band) in the blended films is significantly higher than that in the neat film under the same oxidation conditions. We infer that addition of PC_71_BM enlarges the size of the disordered PffBT4T-2OD in the blend films and thus facilitates generation of LPs. Moreover, we find that the LP-to-DP population ratio in the blended film increases from 0.380 ± 0.015 to 0.440 ± 0.015 after thermal annealing at 80 °C compared to the unannealed film, implying an increase of the PffBT4T-2OD disordered area. Here, we propose a schematic illustration to describe the morphology evolution of the PffBT4T-2OD:PC_71_BM blends after thermal annealing (Scheme 1): After thermal annealing, the originally ordered PffBT4T-2OD around the interface (dotted line region) was disarranged, due to the aggregation of the PC_71_BM around the interface. Similar effect has been reported previously for P3HT/PCBM blends [36]; Meanwhile, according to the TRPL study, the size of the PffBT4T-2OD phase increases from L1 to L2, due to the aggregation of the donor during thermal annealing process.

### 3.6. Carrier Recombination in PffBT4T-2OD:PC_71_BM Blend Films

**Figure 6:**
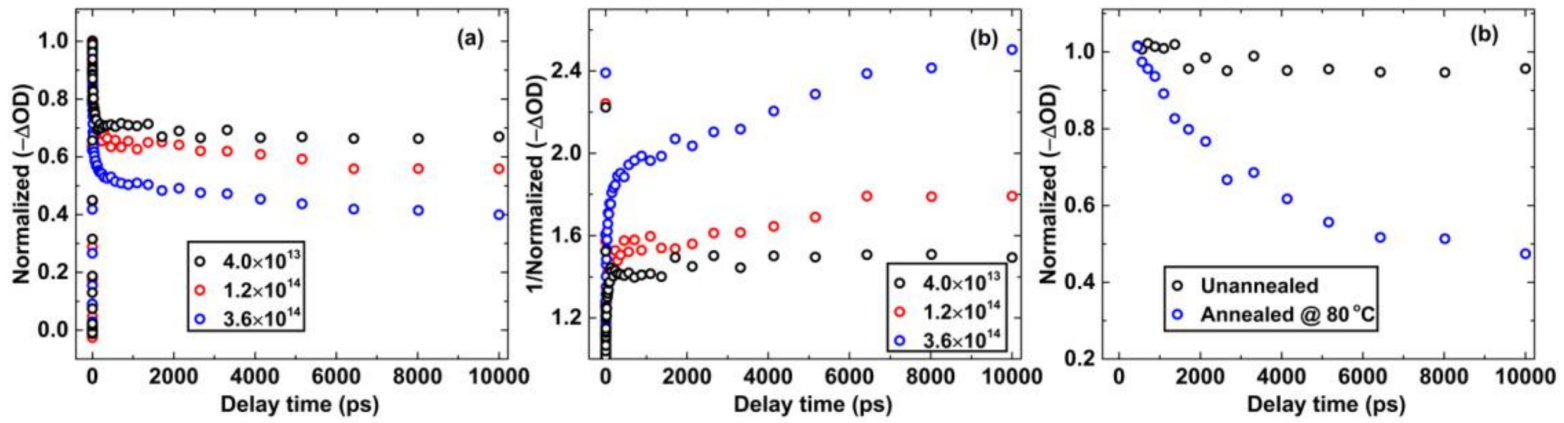
(**a**) Normalized ΔOD and (**b**) 1/(Normalized ΔOD) kinetics of the unannealed PffBT4T-2OD:PC_71_BM film after photoexcitation at 650 nm and probe at 700 nm. The excitation fluencies are 4.0 × 10^13^, 1.2 × 10^14^ and 3.6 × 10^14^ photons/cm^2^, respectively; (**c**) normalized TA kinetics of the unannealed and 80 °C annealed PffBT4T-2OD/PC_71_BM films after photoexcitation at 650 nm under excitation fluency of 4.0 × 10 ^13^ photons/cm^2^.

To understand charge recombination processes of PffBT4T-2OD:PC_71_BM solar cells, we conduct transient absorption measurements. The blend films were excited at 650 nm, and probed at 700 nm. According to the absorption spectra, we excite and probe PffBT4T-2OD absorption bands. At 700 nm, the change of optical density is comprised of ground state bleaching, stimulated emission, excited state absorption and so forth. Many photoexcited processes such as exciton dissociation, charge generation and recombination, can induce the kinetics decay at this wavelength, especially at early delay times. For ease of analysis, we studied the kinetics decay in the range of 500 ps~10 ns dominated by carrier recombination processes (as other processes are most probably over at these delay times). As can be seen from the kinetics of unannealed PffBT4T-2OD:PC_71_BM film in Figure 6a, the population relaxation in 500 ps~10 ns depends on the excitation fluencies, indicating that the decay is caused by bimolecular charge recombination as the decay becomes faster with the increase of the excitation density from 4.0 × 10^13^ to 3.6 × 10^14^ photons/cm^2^. We confirm this via re-plotting of the long-time scale kinetics in the form that is most appropriate for visualization of the second order process, namely with −1/ΔOD plotted versus time (Figure 6b). As expected, this representation clearly demonstrates a linear increase of −1/ΔOD with the slope increasing at higher excitation intensities and therefore higher charge concentrations. This shape of the kinetics is clearly related to bimolecular carrier recombination of PffBT4T-2OD^+^ and PC_71_BM^−^. We can exclude the geminate recombination contribution as it should not depend on the charge concentration [37,38]. Similar phenomenon was also observed in 80 °C annealed PffBT4T-2OD/PC_71_BM films (Supplementary Materials S3). To study the effect of post thermal annealing on carrier recombination processes, we compared TA kinetics of unannealed and 80 °C annealed blends. As shown in Figure 6c, the decay of 80 °C annealed blend is much faster than that of unannealed blend, implying that bimolecular recombination is enhanced after annealing. Considering the morphology evolution in the scheme 1, we infer that photogenerated PffBT4T-2OD^+^ can quickly diffuse away from the interface in the ordered PffBT4T-2OD phases in unannealed blends, because of high carrier mobility in the PffBT4T-2OD crystals [10]. For annealed films, photogenerated PffBT4T-2OD^+^ would diffuse in the disorder PffBT4T-2OD region around the interface first. Thus, the probability of PffBT4T-2OD^+^ to move away from the interface in annealed blend would be lower, which will enhance bimolecular recombination.

## 4. Conclusions

In summary, we have produced PffBT4T-2OD/PC_71_BM solar cells with PCE of 9.5% without any post annealing process and studied the correlations between thermal annealing and charge photogeneration and recombination processes. We found that thermal annealing reduces exciton dissociation efficiency and enhances bimolecular carrier recombination, and both effects should degrade the performance of the PffBT4T-2OD/PC_71_BM solar cells. We associate the enhanced bimolecular charge recombination after thermal annealing with generation of the localized PffBT4T-2OD polarons. 

## Figures and Tables

**Table 1:** The average photovoltaic parameters of neat PffBT4T-2OD and PffBT4T-2OD:PC_71_BM solar cells with the corresponding standard deviations. The parameters were averaged by 5 devices.

Active Layers	*J*_sc_ (mA/cm^2^)	*V*_oc_ (V)	FF	PCE (%)	PCE-Max (%)
PffBT4T-2OD	0.18 ± 0.01	0.55 ± 0.01	0.54 ± 0.03	0.05 ± 0.01	0.06
Annealed PffBT4T-2OD	0.16 ± 0.01	0.67 ± 0.02	0.45 ± 0.03	0.04 ± 0.01	0.05
PffBT4T-2OD:PC_71_BM	21.92 ± 0.29	0.73 ± 0.01	0.60 ± 0.02	9.50 ± 0.23	9.75
Annealed PffBT4T-2OD:PC_71_BM (80 °C)	16.97 ± 0.17	0.75 ± 0.01	0.59 ± 0.02	7.46 ± 0.13	7.65
Annealed PffBT4T-2OD:PC_71_BM (150 °C)	15.32 ± 0.33	0.76 ± 0.01	0.57 ± 0.01	6.62 ± 0.14	6.80

## Supplementary Materials

The following are available online at https://www.mdpi.com/2073-4360/11/3/408/s1, Figure S1: J-V curves of PffBT4T-2OD devices fabricated at varies post thermal-annealing temperatures; Figures S2: J-V curves of PffBT4T-2OD:PC71BM devices fabricated at varies post thermal-annealing temperatures; Figure S3: Normalized TA kinetics of 80 °C annealed PffBT4T-2OD:PC71BM film after photoexcitation at 650 nm and probe at 700 nm. The excitation fluencies are 4.0 × 10^13^, 1.2 × 10^14^ and 3.6 × 10^14^ photons/cm2, respectively; Table S1: Fitting parameters of TRPL kinetics traces in Figures 4; Table S2: Fitting parameters of TA kinetics traces in Figure S3.
