# Supplementary material for: Effect of Post-Thermal Annealing on the Performance and Charge Photogeneration Dynamics of PffBT4T-2OD/PC71BM Solar Cells
Source: Polymers (Basel). 2019 Mar 2;11(3):408. doi: 10.3390/polym11030408 (PMC6473355; doi:10.3390/polym11030408)
Supplement: Supplementary file 1 [file polymers-11-00408-s001.pdf]

Supplementary materials

Effect of Post-Thermal Annealing on the Performance and Charge Photogeneration Dynamics of PffBT4T-2OD/PC_{71}BM Solar Cells

Wei Zhang[1,*], Rong Hu[2,*], Xiaokang Zeng[2], Xiaojun Su[3], Zhifeng Chen[1], Xianshao Zou[3], Jun Peng[1], Chengyun Zhang[1] and Arkady Yartsev[3]

[1] School of Physics and Electronic Engineering, Guangzhou University, Guangzhou 510006, China

[2] Research Institute for New Materials Technology, Chongqing University of Arts and Science Chongqing 402160, China

[3] NanoLund and Division of Chemical Physics, Lund University, Lund 22100, Sweden

[*]Correspondence: wzhang@gzhu.edu.cn; hurong_82@cqwu.edu.cn

S1 Current density-voltage (*J-V*) curves of PffBT4T-2OD and PffBT4T-2OD:PC_{71}BM devices

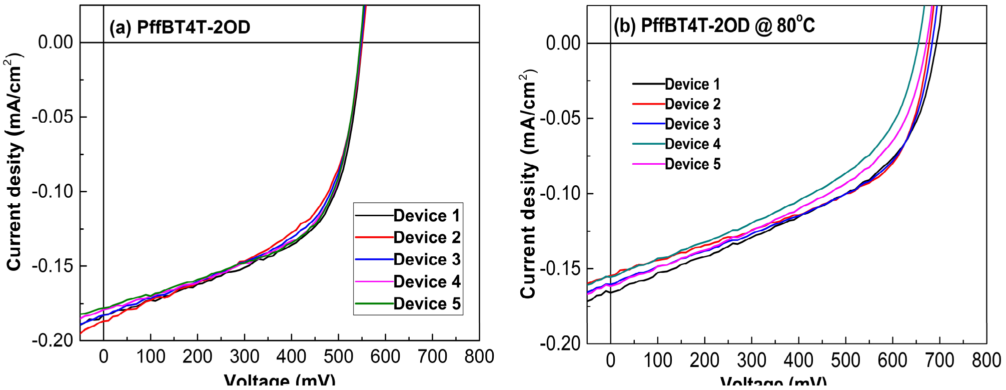

Figure S1. *J-V* curves of PffBT4T-2OD devices fabricated at indicated post thermal-annealing temperatures.

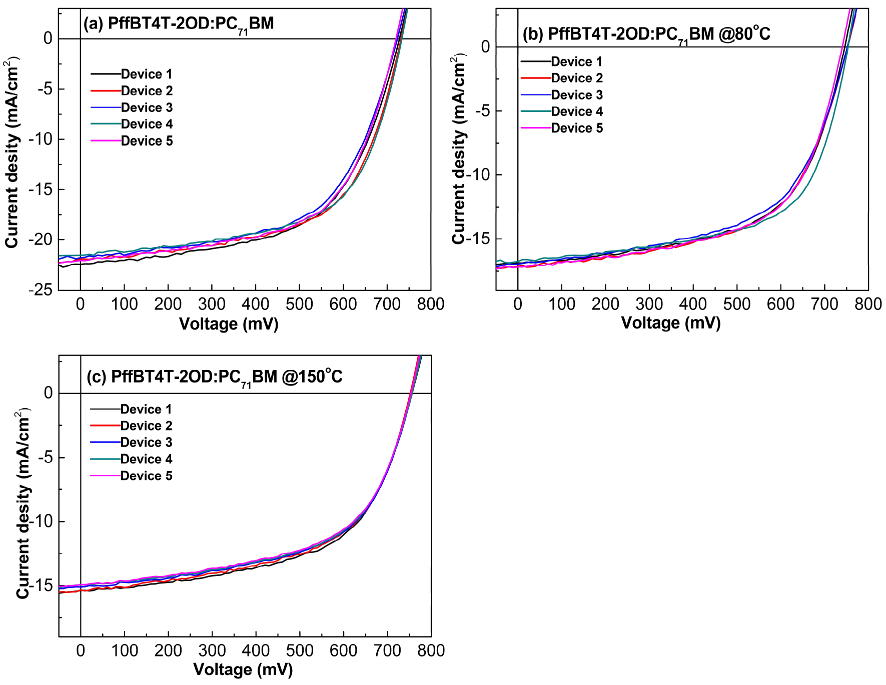

Figure S2. *J-V* curves of PffBT4T-2OD:$PC_{71}BM$ devices fabricated at indicated post thermal-annealing temperatures.

S2 Fitting parameters of TRPL kinetics traces

Table ST1. Fitting parameters of TRPL kinetics traces in Figures 4.

Sample	Fitting parameters				
	A_1	τ_1 (ps)	A_2	τ_2 (ps)	$\bar{\tau}$ (ps)[a)]
Net PffBT4T-2OD	1	305			305
Unannealed PffBT4T-2OD/$PC_{71}BM$	0.34	8	0.66	43	31
80 °C annealed PffBT4T-2OD/$PC_{71}BM$	0.31	9	0.69	83	60
150 °C annealed PffBT4T-2OD/$PC_{71}BM$	0.27	9	0.73	96	72

a) $\bar{\tau} = \frac{A_1\tau_1 + A_2\tau_2}{A_1 + A_2}$

S3 TA kinetics under varies excitation fluencies

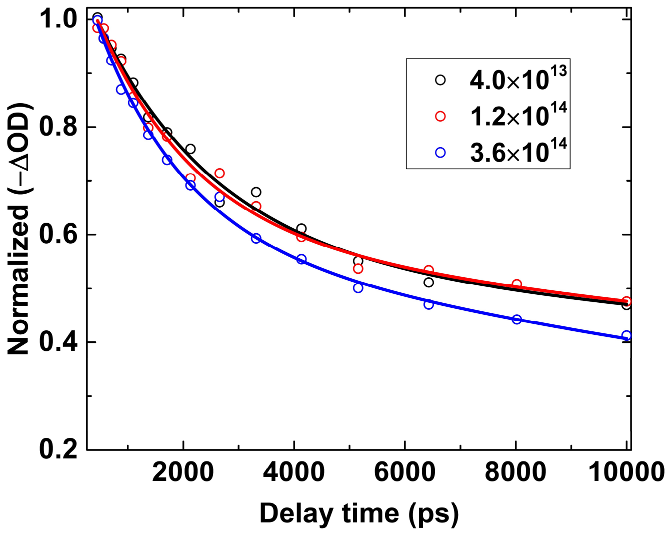

Figure S3. Normalized TA kinetics of 80 °C annealed PffBT4T-2OD:$PC_{71}BM$ film after photoexcitation at 650 nm and probe at 700nm. The excitation fluencies are 4.0×10^{13}, 1.2×10^{14} and 3.6×10^{14} photons/cm^2, respectively.

Table ST2. Fitting parameters of TA kinetics traces in Figure S3.

**Sample**	**Fluence** (photons/cm^2)	**Fitting parameters**			
		A_1	τ_1 (ns)	A_2	τ_2 (ns)
80 °C annealed PffBT4T-2OD/$PC_{71}BM$	4.0×10^{13}	0.13	2.2	0.13	51
	1.2×10^{14}	0.10	1.8	0.11	42
	3.6×10^{14}	0.08	1.6	0.09	25
